# Bootstrapping implementation research training: A successful approach for academic health centers

**DOI:** 10.1017/cts.2021.827

**Published:** 2021-07-26

**Authors:** Kathleen R. Stevens, Elisabeth de la Rosa, Robert L. Ferrer, Erin P. Finley, Bertha E. Flores, Dana A. Forgione, Polly Hitchcock Noel, Timothy A. Reistetter, Melissa Valerio-Shewmaker, Kevin C. Wooten

**Affiliations:** 1 Institute for Integration of Medicine & Science, University of Texas Health Science Center at San Antonio, San Antonio, TX, USA; 2 School of Nursing, University of Texas Health Science Center at San Antonio, San Antonio, TX, USA; 3 School of Medicine, University of Texas Health Science Center at San Antonio, San Antonio, TX, USA; 4 School of Public Health, San Antonio Regional Campus, University of Texas Health Science Center Houston, San Antonio, TX, USA; 5 South Texas Veteran Health Care System, San Antonio, TX, USA; 6 VA Greater Los Angeles Healthcare System, Los Angeles, CA, USA; 7 Texas A&M Corpus Christi College of Business, Corpus Christi, TX, USA; 8 School of Health Professions, University of Texas Health Science Center at San Antonio, San Antonio, TX, USA; 9 Office of the President University of Houston Clear Lake, Clear Lake City, TX, USA; 10 Institute for Translational Sciences University of Texas Medical Branch, Galveston, TX, USA

**Keywords:** Implementation science, research training, translational research, online resources, educational design

## Abstract

Demand for building competencies in implementation research (IR) outstrips supply of training programs, calling for a paradigm shift. We used a bootstrap approach to leverage external resources and create IR capacity through a novel 2-day training for faculty scientists across the four Texas Clinical & Translational Science Awards (CTSAs). The Workshop combined internal and external expertise, targeted nationally established IR competencies, incorporated new National Institutes of Health/National Cancer Institute OpenAccess online resources, employed well-known adult education principles, and measured impact. CTSA leader buy-in was reflected in financial support. Evaluation showed increased self-reported IR competency; statewide initiatives expanded. The project demonstrated that, even with limited onsite expertise, it was possible to bootstrap resources and build IR capacity de novo in the CTSA community.

## Rationale for Novel Curricular Approach

Despite increasing emphasis on implementation research (IR), experts and traditional training opportunities are in short supply [1,2], highlighting the need for innovative training approaches. Bootstrapping refers to a self-starting process to better oneself in the face of limited resources. In a paradigm shift, four (4) Texas CTSAs used this novel approach to create capacity in the face of minimal internal expertise and limited IR resources. We sought to provide and evaluate a high-quality regional training program to increase IR scientific workforce capacity and enhance grant success. This paper discusses how generalizable best education practices and adult education methods in professional training were applied: document the educational need; establish competencies as learning outcomes; provide ongoing educational resources; use active instructional strategies; and determine impact using an evaluation model.

## Unmet Need for Educational Gap

Although IR is not a formalized element of the CTSA Program, it is recognized to be essential in the translational science enterprise to move research into practice and enhance population health [3,4] and is included in the National Center for Advancing Translational Sciences translational science spectrum [5]. As a nascent field, the nature of IR itself has been recast to ensure actionable and relevant findings [6]. This resulted in the emergence of new paradigms, theories, designs, and methods. This fact turns even accomplished researchers into IR novices who seek training in these new methods. Nationally, only half of CTSAs surveyed reported IR training efforts, funded resources, consultation, or research projects; barriers noted were lack of expertise, training, and tools/methods [7]. At the time of the Workshop, no Texas CTSAs housed IR cores/centers, although pockets of expertise existed.

## Target Audience

The Workshop was designed for 30 faculty scientists and clinical partners from Texas CTSA hubs. To assure broad representation, faculty scientists from all four Texas CTSAs were invited. Participants registered in advance and completed pre- and post-Workshop activities. Interest was high and Workshop capacity was expanded. Sixty-three participants represented the four Texas CTSAs, multiple disciplines, institutional and military clinical partners, and research support administrators. Ninety-eight percent of participants held a doctoral degree.

## Description of the Educational Method and Curricular Program

Because terminology in IR is not yet distinct, the planning group developed the following amalgamated definitions to promote common understanding for the initiative:

- Implementation science (IS) is a specialized field that addresses uptake of evidence-based practices into everyday care to improve health and health care. This science is built through IR studies.
- IR builds IS through the scientific investigation of methods and strategies that promote systematic application of research findings in routine clinical practice.

The Community Engagement core at the Institute for Integration of Medicine & Science at University of Texas (UT) Health San Antonio led the design, resourcing, and conduct of an educationally sound 2-day Workshop with its institutional and statewide CTSA partners. Perspectives from multiple disciplines were included in the planning committee.

Workshop planners combined the newly released National Institutes of Health/National Cancer Institute (NIH/NCI) OpenAccess IR training materials [2] with in-person internal and external IR experts to design interactive interprofessional learning activities for maximum impact. Using the NIH/NCI online program as the foundation ensured quality, currency, and generalizability of the Workshop. Funds were used to waive registration fees, support guest speakers travel and honoraria, print Workshop materials, and provide refreshments and lunch for working sessions.

The program was based on an established national curriculum [8], national consensus on dissemination and implementation (D&I) research competencies [9], and recently published NIH/NCI OpenAccess training materials [2], offering a standardized frame. This approach provided two advantages: generalizability beyond the single training opportunity, and alignment with established IR standards to advance the field. The Workshop targeted basic-to-intermediate level competencies with these goals: 1) build interprofessional scientific capacity among teams of faculty and clinical partners to design and conduct relevant, rigorous, high priority studies; 2) enhance success in competing for NIH, Agency for Healthcare Research and Quality, Department of Defense, and Patient-Centered Outcomes Research Institute IR grants; and 3) position UT System research partners as IR leaders for healthcare improvement.

**Fig. 1.**
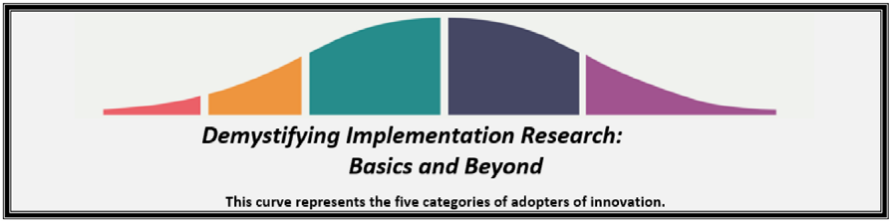
Workshop logo.

To engage faculty scientists, we employed dissemination strategies [10] and marketing approaches. A logo “branded” the Workshop made materials quickly identifiable and created a shared mental model of Workshop purpose, with “adoption” as the core idea. Initial logo designs were vetted by the planning group. The final logo (Fig. 1) contained built-in meaning, depicting “adoption.” [11]

Measurable learning objectives (Table 1) aligned with the NIH/NCI curriculum and guided selection of content, topics, and speakers (see details at https://iims.uthscsa.edu/sites/iims/files/Comm_Engagement/Workshop%20Report%20FINAL.pdf).

**Table 1.** Learning objectives guiding the training Workshop curriculum

Assemble sufficient evidence of clinical intervention effectiveness and appropriate fit for a given clinical context Explain the evolution, current state, and future agenda of implementation science and its value to population health Define outcome measures for both implementation strategy (system outcomes) and clinical intervention (patient/population outcome) Select conceptual models and theoretical justification to support the choice of implementation strategy and inform the design, variables to be measured, analytic plan, and sustainment Describe implementation strategies for moving evidence into practice including existing taxonomies/classification schema State a research question addressing a gap in the provision of an evidence-based intervention, practice, or policy Summarize study designs used in implementation research and their relative strength Describe key elements in forming a business plan for sustainment, identifying implementation costs and quantifying benefits Apply principles of the “science of team science” to enhance productivity of multidisciplinary study teams and achieve adaptive implementation and sustainable change Outline an engagement process that will gain support from relevant stakeholders to ensure feasibility of the study plan Draft a prospectus targeted at a D&I funding opportunity from a variety of agencies

We applied proven instructional design principles [12,13] to achieve the learning objectives. Activities included presentations/Q&A, guided individual and group work, panel discussions, and online resources to accommodate adult learner preferences. Learning strategies included pre-Workshop assignments and in-person interactive group activities. The availability of previously developed online IR training resources enabled participants to prepare for the 2-day experience. Pre-Workshop activities (Table 2) and resources were emailed to participants, and they were encouraged to complete these activities as a condition for registration fee waiver.

**Table 2.** Pre-class assignments: “5 things to do before you come”

Complete the pre-Workshop *Self-Assessment of Competencies in Dissemination & Implementation Sciences* [9] Review the ImpRES Tool [14] and have a copy handy on your electronic device View *Orientation to the Science of D&I* from NIH/NCI Use the 6 *OpenAccess NIH/NCI TIDIRC* modules to support work prior, during, and following the Workshop Save the following videos and readings (adapted from NIH/NCI TIDRC*) in your resource file for continued use Module 1: *Intro. to D&I* – Dr. Russ Glasgow, Univ. of Colorado School of Medicine, Denver (32 mins.; view before Workshop) Module 2: *Fidelity & Adaptation* – Dr. David Chambers, NCI (38 mins.) Module 3: *Overview of D&I Models* – Dr. Wynne Norton, NCI (26 mins.) Module 4: *Intro.to D&I Measures* – Dr. Cara Lewis, Kaiser Permanente (35 mins.) Module 5: *D&I Designs Overview* – Dr. Greg Aarons, Univ. of California San Diego (65 mins.) Module 6: *Implementation Strategies* – Dr. Prajakta Adsul, NCI (19 mins.)

* As of December 2019. See NIH updates June 10, 2021.

In tandem with pre-Workshop assignments, in-person activities were employed. Because team-based learning (TBL) (an active, learner-centered but instructor-led strategy) is shown to enhance learning [15,16], we scheduled group activities throughout the Workshop. Groups applied a step-by-step research design guide [14] to co-develop study ideas. TBL strategies were used to expand and reinforce learning, promote interprofessional collaboration, and move toward the development of IR project ideas.

Participants received a comprehensive four-color hardcopy workbook which included the syllabus, schedule, speaker biographies, worksheets, IR resources, participant list, and evaluation link. An electronic file (PDF) of the workbook was provided to registrants before the Workshop. Additionally, speakers shared presentation slides, helpful supplemental resources, and readings for their respective session. These were saved in a shared Google Drive folder for availability during and after the Workshop.

Interspersed summaries and participant-panel discussions stimulated ideas for collaboration, culminating in recommendations for next steps. To conclude the Workshop and focus on long-range capacity building, one representative from each Texas CTSA hub moderated a panel discussion entitled *Building Scientific Workforce Capacity and Opportunities for Collaboration.* This brainstorming session provided opportunity for participants to generate ideas and recommendations for preferred strategies to continue expansion of IR across the CTSAs.

## Methods of Evaluation

To assess near-term impact and long-range sustainment of IR capacity building among Texas CTSAs, a comprehensive evaluation plan was designed. The Kirkpatrick model [17], widely used in evaluating training programs, provided structure. Table 3 identifies the four levels associated with Workshop outcomes and types of data gathered.

**Table 3.** Evaluation plan

Kirkpatrick level [17] and question answered	Workshop outcome
Level 1 Reaction *Was the training engaging, favorable, and relevant?* *Were the objectives covered?*	Participant rating of Workshop experience
Level 2 Knowledge Acquisition *What knowledge, attitudes, and confidence were gained?*	Participant pre-post-Workshop ratings of their D&I competencies
Level 3 Behavior *Is the knowledge gained applied in participants’ jobs?*	Action Plan for an IR study Identification of potential investigative team
Level 4 Results *What is the impact of the training on targeted outcomes?*	Ongoing collaboration across Texas CTSAs Submission of D&I grants Grants won

## Results

### Institutional Support

The formal value proposition stimulated leadership buy-in for faculty involvement in this new research focus and garnered sufficient funds to support the costs of the Workshop. The four-hub planning approach raised interest and catalyzed institutional support of faculty-scientist participation and travel from each hub.

### Impact of Training

Results from Workshop quantitative and qualitative data were classified into each of the four Kirkpatrick levels and analyzed.

#### Level 1 Workshop Experience

Twenty-seven of the 63 participants (42%) completed both the pre- and post-Workshop self-assessment, which included evaluating whether the 11 learning objectives were met (Table 1). The highest rated objective was #2 *Evolution of IR* (96% agreement); the lowest was #11 *Prospectus Plan* (59% agreement). Event location was rated excellent (63%) or good (33%). Participants reported networking opportunities as excellent (52%), good (33%), and fair (7%). Survey comments indicated that the Workshop experience was viewed very favorably. Comments included, “Wonderful workshop, thank you for inviting exceptional speakers and planning content that could help us immediately!” Also, “This workshop has made me think about how we build teams and projects. In science we are oriented to strive for the PI role but serving as a co-investigator is just as important.”

#### Level 2 Knowledge Acquisition

In the pre-post self-assessment of IR competencies, participants reported improvement across all domains of IR competencies (IR definitions, background/rationale, theory/approaches, design/analysis, and practice-based considerations). After the training, fewer participants (48%) considered themselves “novice” than prior to the training (80%). Also, the proportion of “Intermediate/advanced” participants increased from 25% to 52%. Familiarity with the IR theories increased from 56% to 73%. Participant response to open-ended questions indicated that the Workshop had a positive impact on knowledge and attitudes. One example was, “This program and workshop have provided me huge takeaways.”

#### Level 3 Behavior

In the group activities, participants connected with colleagues with similar interests. Groups used the ImpRES Tool [14] to develop ideas for an IR study. A participant stated, “This workshop has made me rethink how to use mixed methods in Implementation Science.” Another stated, “This workshop has taught me that I should also map to a theory and model and consider the inner setting.” A third commented, “Going forward, how can we make people aware of each other to allow for the development of natural collaborations?”

#### Level 4 Results

Participants indicated interest in ongoing network interaction, resource availability, and capacity building including consultation. One person commented, “Overall, I consider this to be a successful workshop because we have laid groundwork for collaborations that could lead to possible grant opportunities.” Another participant offered, “A network of clinical sites in Texas could serve to build scientific capacity. Since IR requires multiple sites, the suggestion is to develop formal collaboration across the Texas CTSAs.”

Participants endorsed first steps toward establishing the statewide Texas Implementation Science Research Network (TEX-IS). Given that the Texas CTSAs routinely gather quarterly in regional meetings, this venue was suggested for continuing interaction. Unfortunately, meetings were suspended due to the pandemic. However, ongoing communication has been achieved through the monthly *TEX-IS Research Network New Notes* e-newsletter.

#### Progress subsequent to the Workshop

Progress 12 months following the Workshop includes:

- Posting Workshop learning resources on the host institution’s website https://iims.uthscsa.edu/community/activities.html
- Launching a D&I Consultation Core to support investigators and healthcare professionals in integrating D&I science in their research and practice. (https://reach.uthscsa.edu/services/di/). As a direct result of the show of interest in IR, new Implementation Research Consultation Core was established at one hub.
- Offering a series of UT Health Houston IR webinars (https://sph.uth.edu/research/centers/chppr/workshops/tiis/conference).
- Publishing monthly newsletters of emerging trends in IR, including new resources on equity in IS (e.g., 2). To date, the *TEX-IS Research Network News Notes* has published eight monthly issues.
- Creating the TEX-IS Research Network Listserv and registration site.

## Discussion

Workshop program design and curriculum were key deliverables of this project. A key element for success was using the NIH/NCI curriculum: Planners capitalized on the newly released NIH/NCI OpenAccess training resources, providing opportunity for ongoing and expanded training to additional faculty scientists across the four UT System CTSAs. Similarly, other notable online resources [1] that align with the national training curriculum can also be leveraged.

While this test case focused on IR, the educational principles we used are applicable to a wide range of capacity building needs in translational science. Developers of such programs can consider interdisciplinary planning, articulated program goals, measurable learning objectives, active instructional strategies, extended resources, ongoing interaction, and formal evaluation approach. The 2-day curriculum and learning activities provided a comprehensive foundation for extending IR training initiatives throughout our region.

Limitations arise from several sources. First, this training program was offered throughout the four CTSAs hubs in Texas, each with geographical differences. CTSA hub similarities likely arose from the overarching UT System (state-wide) context. Also, regional differences may result in different responses from single hub in faculty participants and populations impacted. Future work could include demographic composition of attendees to throw light on equity. Although measures are in place to promote multi-site IR studies, the reality of the intervening pandemic changed the intended dynamics of collaboration. Finally, although the program is educationally sound, it is a single test case of a novel approach to bootstrapping resources to boost IR workforce capacity.

The program incorporated a standardized foundation for IR training to advance our capacity in IR effectively and efficiently. Our model combines internal strengths with external resources (both in-person experts and online learning resources) and provides a comprehensive plan for building and evaluating workforce capacity, beginning with the 2-day Workshop.

The project demonstrated that, even with limited onsite expertise, it was possible to bootstrap resources and build IR capacity de novo in the CTSA community. Our approach could be useful to other institutions to expand IR capabilities in the face of limited local expertise.
